# Improved Metal Oxide Electrode for CIGS Solar Cells: The Application of an AgO_*X*_ Wetting Layer

**DOI:** 10.1186/s11671-021-03506-1

**Published:** 2021-03-20

**Authors:** Nils Neugebohrn, Norbert Osterthun, Maximilian Götz-Köhler, Kai Gehrke, Carsten Agert

**Affiliations:** DLR Institute of Networked Energy Systems, Urban and Residential Technologies, Carl-von-Ossietzky-Str. 15, 26129 Oldenburg, Germany

**Keywords:** Transparent conductive electrode, Ultrathin Ag film, Wetting layer, AgO_*X*_, Oxide–metal–oxide, Front contact, Building integrated photovoltaics, Copper indium gallium selenide

## Abstract

Oxide/metal/oxide (OMO) layer stacks are used to replace transparent conductive oxides as front contact of thin-film solar cells. These multilayer structures not only reduce the overall thickness of the contact, but can be used for colouring of the cells utilizing interference effects. However, sheet resistance and parasitic absorption, both of which depend heavily on the metal layer, should be further reduced to reach higher efficiencies in the solar cells. In this publication, AgO_*X*_ wetting layers were applied to OMO electrodes to improve the performance of Cu(In,Ga)Se_2_ (CIGS) thin-film solar cells. We show that an AgO_*X*_ wetting layer is an effective measure to increase transmission and conductivity of the multilayer electrode. With the presented approach, we were able to improve the short-circuit current density by 18% from 28.8 to 33.9 mA/cm^2^ with a metal (Ag) film thickness as low as 6 nm. Our results highlight that OMO electrodes can be an effective replacement for conventional transparent conductive oxides like aluminium-doped zinc oxide on thin-film solar cells.

## Introduction

Oxide/metal/oxide (OMO) electrodes are able to substitute transparent conductive oxides (TCO) like indium tin oxide or aluminium-doped zinc oxide (AZO), which are typically used as electrodes in a large variety of devices including light-emitting diodes, displays, touch screens and photovoltaic modules. Key aspects of OMO electrodes that arise from their reduced thickness are the short deposition time and better mechanical flexibility. That makes them cheaper to produce and a robust alternative to TCOs, while providing equivalent or superior optical and electrical characteristics [1, 2]. The fact that comparable or better results can be achieved with OMO electrodes on solar cells than with conventional AZO electrodes has already been demonstrated earlier using the example of amorphous silicon thin-film solar cells [3]. Furthermore, due to a low deposition temperature OMO electrodes are suitable for temperature sensitive devices like organic photovoltaics or polymer substrates [1, 2].

**Fig. 1:**
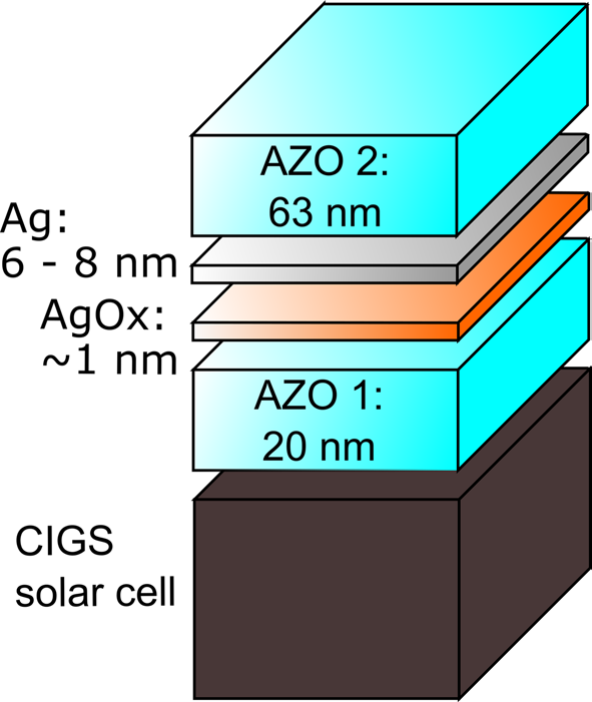
Schematic layer stack of OWLMO electrode on the CIGS solar cell. The conductivity and transparency of the intermediate Ag layer can be improved on with a wetting layer

Most interestingly, OMO electrodes act as optical cavities due to the interference caused by multiple reflections at the layer interfaces. This allows designing the electrode to possess a broad peak of very high transmission despite the use of a highly reflective metal layer [1, 3]. By employing a metal with a low refractive index and therefore a high reflectivity, the strength or finesse of the optical cavity is increased and so is the transmission in the resonance region [4]. The spectral positions of the transmission and reflection peaks are determined by the optical thickness of the oxide layers, while the electrical conductivity is mainly influenced by the metal film. Therefore, it is possible to tune the optical characteristics of the cavity separately from the electrical ones. This allows not only to design the electrode according to the electrical and optical requirements of different photovoltaic absorbers or cell technologies, but also to use it in a multifunctional manner. It has been shown that special optical properties of OMO electrodes can be used for the colouring of PV modules [5–7]. We previously applied OMO electrodes with integrated colouring on CIGS thin-film solar cells, which are an attractive option for modules specifically designed for building integration [5]. One huge challenge in the development of OMO electrodes is the deposition of the ultra-thin metal layer (< 15 nm) sandwiched between the two oxide layers. Here, typically silver (Ag) is used, as it has the lowest resistivity of all metals [8]. Ideally, the Ag film has to be as thin as possible for the highest transmission with minimal absorption losses. Therefore, theoretically a lower limit is set only by the desired conductivity. However, due to significant dewetting of Ag, a three-dimensional Volmer–Weber-type island growth is observed for Ag layers with thicknesses below the percolation threshold (*d*_pt_) of about 10 nm [2, 9–12]. The transmittance at these low thicknesses is severely limited by the absorbance and scattering due to surface plasmon resonances occurring at the metal clusters [2, 13, 14]. Moreover, the formation of islands leads to an increased resistivity [15, 16]. A transition to a fully closed, continuous film can be observed with increasing metal thickness above the percolation threshold *d*_pt_. This is accompanied by a reduction in resistivity, as well as an increase in the transmission peak height, though transmission decreases again for layers surely thicker than the percolation thickness *d* > *d*_pt_ [2, 12, 17]. The value of *d*_pt_ is related to the surface free energies of the deposited metal, the substrate and the interface between them [18]. Various strategies have been proposed and reviewed in detail to reduce *d*_pt_ and to achieve a flat Ag layer with full coverage by increasing the adhesion to the substrate or decreasing the surface free energies of the metal or the interface. They include alloying of different metals [19, 20] or the addition of gases during deposition of the Ag layer [10, 12]. Furthermore, a number of different wetting layers have been studied to improve the quality of thin Ag layers, including Ge, AgO_*X*_ and Cu [2, 17]. Ge was found to provide the best wettability, but the optical losses due to strong absorption of Ge make it a bad choice for optical applications [17]. In particular, AgO_*X*_ has shown promising results. Zhao et al. found percolation thicknesses of 6 nm and 8 nm for Ag layers with and without AgO_*X*_ wetting layers in OMO electrodes [17], and comparable results were achieved by H. Jo et al. and W. Wang et al. with full AgO_*X*_ films [10, 12]. AgO_*X*_ has the advantage that the deposition is easily implemented in the OMO process by adding oxygen as a reactive gas. Furthermore, an AgO_*X*_ wetting layer (WL) is preferable to full AgO_*X*_ layer, as the higher refractive index of AgO_*X*_ compared to pure Ag would reduce the strength of the optical cavity due to a smaller refractive index difference with the AZO [11, 12]. While OMO electrodes have been used for Cu(In,Ga)Se_2_ (CIGS) solar cells previously [5, 21], the effect of WL on OMO/CIGS solar cells has not been studied yet. In this publication, we demonstrate the impact of an AgO_*X*_ wetting layer on an OMO used as the transparent front electrode of CIGS solar cells. We show that the photocurrent together with the efficiency of CIGS cells can be significantly increased by using OMO electrodes with AgO_*X*_ wetting layer, compared to conventional OMO layer stacks (Fig. 1).

## Materials and Methods

Reference oxide/metal/oxide (OMO) electrodes were prepared via DC magnetron sputtering at room temperature as described previously [5]. The bottom and top AZO layers of the OMO electrode have a thickness of 20 nm and 63 nm and were deposited with an oxygen flow of 0 sccm and 5 sccm, respectively. The oxide/wetting-layer/metal/oxide (OWLMO) electrodes, as shown in Fig. 1, were achieved by the deposition of an additional AgO_*X*_ wetting layer after the first oxide by sputtering of Ag with 45 sccm Argon and 10 sccm oxygen at 0.8 Pa and 200 W at room temperature for one second. We assumed the deposition rate of AgO_*X*_ to be equal or slightly less compared to that of pure Ag, as it is known for reactive sputtering. Therefore, in the following, the wetting layer thickness is estimated to be 1 nm and a wetting layer followed by e.g. 6 nm pure Ag will be described with a total thickness of 7 nm. Deposition rates of the Ag and AZO sputter processes were determined with a Veeco Dektak 150 profilometer. The thicknesses of the layers of the samples as described in this study are based on these deposition rates, which were 1.03 ± 0.08 nm/s for Ag, 1.41 ± 0.02 nm/s for AZO with 0 sccm oxygen flow and 1.38 ± 0.01 nm/s for AZO with 5 sccm oxygen flow. The CIGS cells used are based on state-of-the-art, Cd-free CIGS processes by AVANCIS, leading to aperture efficiencies of up to 19% on small modules [22]. In order to enable the application of the alternative front contact, these cells were modified to provide a suitable test cell for this study. Specifically, instead of a complete removal of the standard front contact AZO its thickness was reduced to about 200 nm to keep the optimized interface of the state-of-the-art cells. Additionally, this has the benefit of increased stability and protection of the cells during transport and increases the reproducibility of our experiments. The CIGS samples have an overall size of 2.5 × 2.5 cm^2^, from which nine 0.25 cm^2^ cells were created by mechanical scribing. Reflection spectra of the OMO/CIGS samples were recorded with an UV–VIS Cary 5000 spectrophotometer with an integrating sphere. The sheet resistance of the OMO/CIGS samples was measured with a Jandel RM3-AR four-point probe system. Due to the brittle nature of CIGS, non-optimal contacting (e.g. puncturing to the back contact) can lead to outliers in sheet resistance values. Therefore, the median instead of average sheet resistance values was used for evaluation. 15–20 measurements were taken to determine the median sheet resistance of each sample. In order to evaluate the cell performance, current–voltage measurements were performed with a WACOM dual lamp solar simulator according to standard test conditions (AM1.5G-spectrum, 1000 W/m^2^, 25 °C). The system has a relative error of the efficiency of 1.13% including the reference cell error, measurement device error and the power fluctuations of the irradiation. The external quantum efficiency (EQE) was recorded with an RR-2100 measurement system from LOT Oriel.

## Results and Discussion

**Fig. 2:**
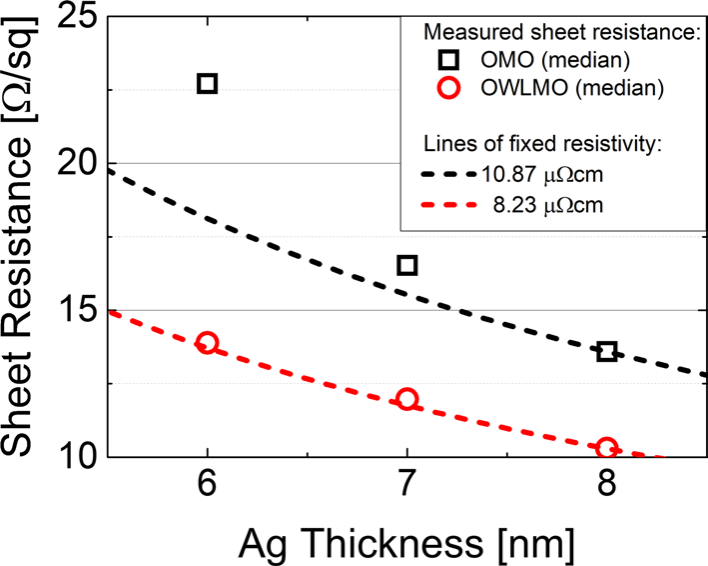
Comparison of the sheet resistance measured of OMO electrodes of the CIGS solar cells with different thicknesses of the intermediate metal. Samples without (black) and with (red) wetting layers are shown. The dashed lines represent the sheet resistance of OMOs with constant resistivity, as would be expected without a change in morphology of the Ag layer

**Fig. 3:**
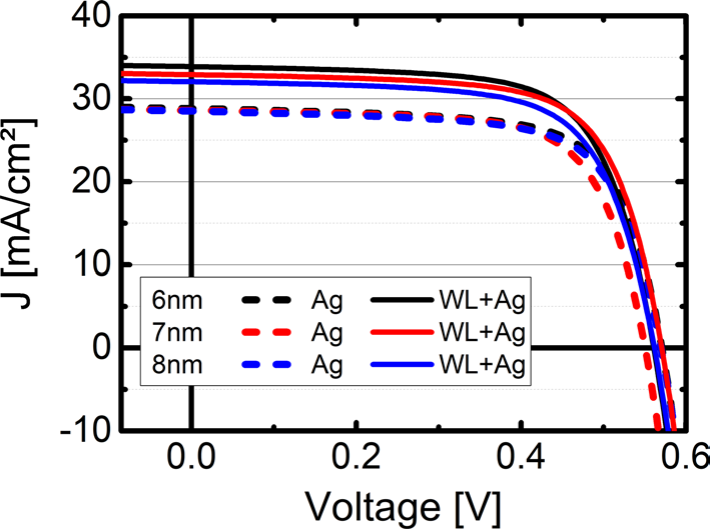
Current density–voltage characteristics of CIGS cells using electrodes with (full lines) and without (dashed lines) wetting layers for the three thickness levels of the Ag layer are compared. The best performing cell from the set of nine cells structured on each sample is shown

**Fig. 4:**
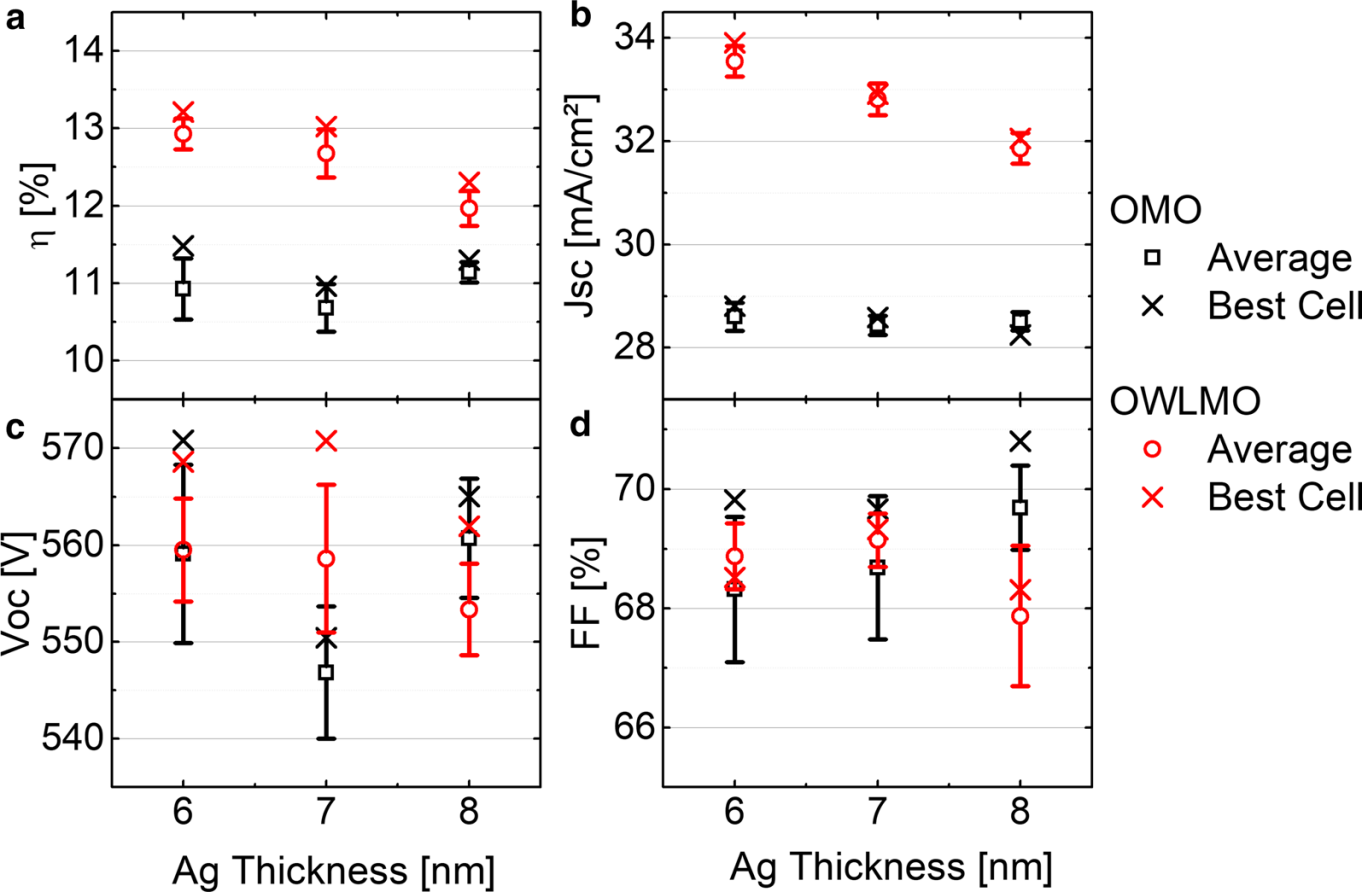
Comparison of the cell performance parameters efficiency (**a**), short-circuit current density (**b**), open-circuit voltage (**c**) and fill factor (**d**). The open symbol indicates the average of a set of nine cells and the cross the value of the best performing cell

**Fig. 5:**
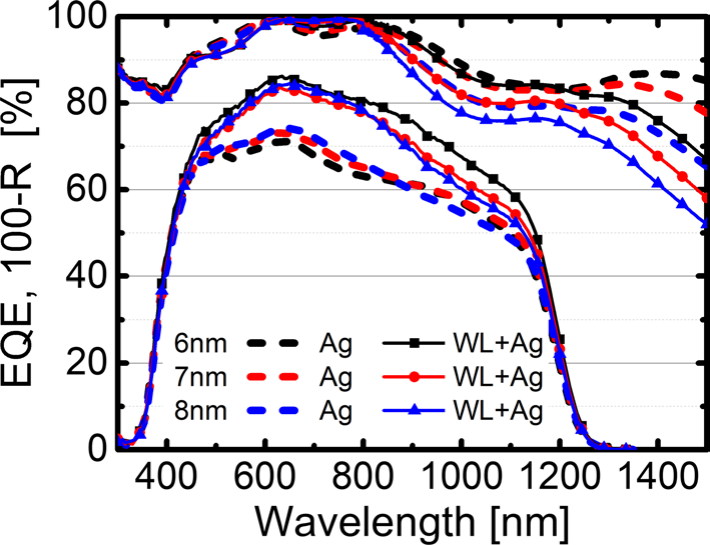
The external quantum efficiency (EQE) and the reflectance subtracted from 100% (100%-R) of CIGS cells using OMO electrodes with (full lines) and without (dashed lines) wetting layers for the three thickness levels of the Ag layer are shown. The best performing cell from the set of nine cells structured on each sample is shown

The electrical performance of the samples with (OWLMO) and without (OMO) wetting layer was evaluated by their resistive characteristics. In Fig. 2, the sheet resistance of the samples is shown. Prior to the deposition of OMO electrodes, a sheet resistance of 56 ± 3 Ω/sq was measured for the partial front contact of the CIGS solar cells. As expected, the sheet resistance decreases with increasing Ag thickness for OMO as well as OWLMO samples, though the OWLMO samples clearly exhibit a lower overall sheet resistance. However, a comparison to a material with constant resistivity (dashed lines) shows that only the resistivity of the OWLMO electrodes follows the expected trend. The sheet resistance of the OMO electrodes rises much stronger for thinner Ag layers than expected for a constant resistivity. This indicates that for the OMO samples, Ag layers with thickness below 8 nm are not fully closed due to Volmer–Weber-type island growth, while the AgO_*X*_ wetting layer in the OWLMO samples is able to suppress this dewetting behaviour. The sheet resistance for OWLMO samples is not only lower than for OMO samples, but a nearly constant resistivity of about 8.2 µΩcm is achieved for all thicknesses. This indicates that due to the wetting layer, a high degree of coverage of the Ag film can be reached even for thicknesses as low as 6 nm (WL + Ag). Furthermore, the sheet resistance of 13.9 (10.3) Ω/sq with 6 (8) nm WL + Ag achieved here is in good agreement with the one reported by G. Zhao et al. with 12.5 Ω/sq on PET substrates [17]. On CIGS cells, the substrate employed in this publication, Kang et al. published a sheet resistance of 104 Ω/sq for their best performing cell with an OMO electrode employing a Cu–Mo metal layer [21]. Even further improvement of OMO electrodes employing Ag might be possible, as bulk Ag has a resistivity of only 1.6 µΩcm [8],

In Fig. 3, the JV characteristics of the cells with and without OWLMO layers are shown. The samples including a wetting layer show an increased current density of up to 5 mA/cm^2^ compared to the reference OMO electrodes. Furthermore, the samples with an OWLMO electrode show a decrease in the current density with increasing Ag thickness, while the current density of samples with an OMO electrode does not change over the range of 6–8 nm Ag. In Fig. 4, this trend is also clearly visible in the plot of the short-circuit current density J_SC_ over thickness, though no clear impact on open-circuit voltage or fill factor is apparent. A decrease in current density with increasing metal (Ag) thickness is to be expected due to the increased reflectance of a thicker metal layer. However, due to the small sample size of 0.25 cm^2^, no significant impact of the sheet resistance on J_SC_ is present. In Fig. 5, the EQE of each sample is shown together with the respective reflectance. The EQE measurements confirm the results from the current–voltage characterization. OWLMO layers and OMO layers can be clearly distinguished from each other. The wetting layer improves the quantum efficiency by up to 17% in a wavelength interval of 400–1200 nm. A slight decrease of about 2% in reflection can be observed at 710 nm. However, it is not enough to explain the increase in EQE. Furthermore, the reflection and the EQE increase for wavelength above 800 nm with the introduction of the wetting layer. Therefore, it can be concluded that the transmittance of the OWLMO electrode is improved due to reduced absorption in the Ag layer. The short-circuit current density calculated from the integrated EQE is also in good agreement with the results of the JV measurement (Table 1).

**Table 1:** Short-circuit densities as calculated from EQE and JV measurements are in good agreement

Ag thickness (nm)	OMO J_SC_ (mA/cm^2^)		OWLMO J_SC_ (mA/cm^2^)	
	JV curve	EQE	JV curve	EQE
6	28.81	28.44	33.90	34.14
7	28.58	29.03	32.91	32.60
8	28.24	28.89	32.05	32.51

As previously mentioned, Fig. 4 shows that the J_SC_ of the OMO samples without wetting layer is not influenced by the Ag layer thickness. In the EQE results in Fig. 5, we can see that this is due to the decrease in the EQE in the long-wavelength range being compensated by an increase for shorter wavelengths in the visible range. This can be attributed to an effectively improved quality of the Ag layer with increasing thickness due to the increased coverage and reflectivity of the Ag film. This enhances the finesse of the optical cavity set up by the OMO stack, which is tuned to increase transmittance in the visible range [5]. According to the same argument, the reflectivity of OMO samples for wavelengths above 800 nm increases with Ag thickness, resulting in a reduced EQE in that spectral range.

For OWLMO samples, the effect of higher reflection with increasing Ag thickness at higher wavelength is even more pronounced. Comparing OWLMO to OMO samples, both, the increase in reflectivity for > 800 nm and the reduction in absorption for 400–1200 nm indicate that a more favourable morphology of the Ag layer was achieved due to the wetting layer.

**Fig. 6:**
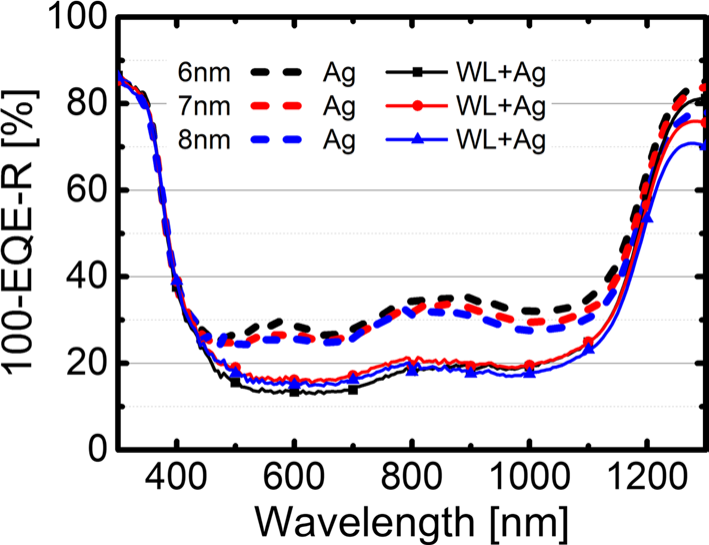
100-EQE-R, i.e. parasitical absorption and recombination losses of CIGS cells using OMO electrodes with (full lines) and without (dashed lines) wetting layers. After subtraction of both EQE and reflection from 100%, only the absorption not contributing to current generation remains

In Fig. 6, the non-radiative recombination losses und parasitic absorption are shown. It is clearly visible that the use of wetting layers reduces the parasitical absorption of the front contact. As discussed previously, we attribute this to an improved homogeneity and coverage as well as a decreased percolation thickness and roughness of the Ag layer. These changes to the Ag layer result in a lower absorption, as was observed previously using other substrates [12, 17].

Despite the improvement in the OMO front contact, the efficiency of about 13% achieved in this study is below that of current state-of-the-art CIGS module from Avancis with 19% [22]. However, since the OMO electrode can be used primarily to influence the current generation, a comparison of the short-circuit current density is more useful. With 34 mA/cm^2^ achieved in this study compared to the 36.3 mA/cm^2^ in the literature, the OMO technology shows its competitiveness even before optimisation of the entire cell stack [22].

## Conclusion

AgO_*X*_ wetting layers have been investigated in oxide/metal/oxide front contacts on CIGS solar cells, regarding an improvement in the short-circuit density and overall efficiency. A decrease in sheet resistance of from 22.71 to 13.89 Ω/sq as well as an increase in short-circuit current density from 28.8 to 33.9 mA/cm^2^ for an Ag thickness of 6 nm was achieved. The results indicate that a significant lowering of the percolation thickness of the Ag films due to the wetting layers was successful, resulting in a lower parasitical absorption by the electrode. The increase in Ag film quality observed here due to the addition of the wetting layer, namely a higher transmission and conductivity, is in good agreement with previous findings in literature. Based on these results, it can be concluded that the wetting layer was successfully implemented for OMO electrodes applied on CIGS thin-film solar cells. The results demonstrate that wetting layers are a valuable addition to improve OMO contacts for solar cell applications.

## Data Availability

The datasets used and/or analysed during the current study are available from the corresponding author on reasonable request.

## Abbreviations

OMO: Oxide/metal/oxide
CIGS: Cu(In,Ga)Se2
AZO: Aluminium-doped zinc oxide
TCO: Transparent conductive oxide
*d*_pt_: Percolation threshold
WL: Wetting layer
OWLMO: Oxide/wetting-layer/metal/oxide
EQE: External quantum efficiency
